# Comparison of the virulence and transmissibility of canine H3N2 influenza viruses and characterization of their canine adaptation factors

**DOI:** 10.1038/s41426-017-0013-x

**Published:** 2018-03-07

**Authors:** In-Won Lee, Young-Il Kim, Gyo-Jin Lim, Hyeok-Il Kwon, Young-Jae Si, Su-Jin Park, Eun-Ha Kim, Se Mi Kim, Hiep Dinh Nguyen, Min-Suk Song, Young-Ki Choi

**Affiliations:** College of Medicine and Medical Research Institute, Chungbuk National University, 12 Gaeshin-Dong Heungduk-Ku, Cheongju, KS001 Republic of Korea

## Abstract

Recent canine influenza outbreaks have raised concerns about the generation of pathogenic variants that may pose a threat to public health. Here, we examine avian-like H3N2 canine influenza viruses (CIVs) isolated from 2009 to 2013 in South Korea from dogs. Phylogenetic analysis revealed that these viruses are closely related to strains previously isolated from dogs in Korea and China. However, molecular characterization demonstrated non-synonymous mutations between the canine viruses, particularly in the putative H3 antigenic sites, NA stalk regions, and in the internal genes of the 2012–2013 isolates compared with the 2009 isolate. Animal experiments showed that three representative isolates (A/canine/Korea/AS-01/2009(AS-01/09), A/canine/Korea/AS-05/2012(AS-05/12) and A/canine/Korea/AS-11/2013(AS-11/13), were readily droplet transmitted between dogs, whereas AS-05/12 induced more severe clinical disease and was lethal in dogs compared with AS-01/09. Although all viruses were able to infect ferrets, AS-05/12 consistently yielded higher nasal wash titers and was transmissible to ferrets via airborne droplets. Using reverse genetics, we show that the NA, NP, and M genes of CIV are critical for the adaptation of avian H3N2 viruses, and the resulting reassortant genotypes promote viral growth in dogs in a manner similar to that of the wild-type AS-01/09 virus. Taken together, these results demonstrate that CIVs continuously evolve in dogs thereby allowing them to gain a foothold in mammalian hosts. Importantly, we elucidated the genetic contributions of the NA, NP, and M genes to the adaptability of CIVs derived from the avian H3N2 virus.

## Inrtoduction

The influenza A virus belongs to the family *Orthomyxoviridae* and contains eight single-stranded, negative-sense RNA segments that encode more than 15 viral proteins^1^.

Influenza A viruses cause perennial epidemics and occasional pandemics in humans and have been implicated in local and worldwide poultry outbreaks that result in significant economic losses^2^. Based on differences in the antigenicity of the hemagglutinin (HA) and neuraminidase (NA) surface viral antigens, 18 HA and 11 NA subtypes have been identified in migratory waterfowl, the natural host reservoir of influenza A viruses^3^. However, microevolutionary processes can lead to significant genetic evolution resulting in the transmission of these viruses to species other than birds^4^.

Dogs are susceptible to influenza A viruses, and in the United States an intact equine-origin H3N8 virus caused respiratory disease outbreaks among racing greyhounds between 2004^5^ and 2006^6^. The causative canine influenza virus (CIV) was also observed in non-greyhound dogs in shelters in the United States^5,6^. In addition, a genetically-related equine-like H3N8 virus was isolated in English foxhounds in the United Kingdom^7^, and sporadic canine infections with the highly pathogenic avian H5N1^8^ and the swine-origin A (H1N1) pdm09^9^ viruses have been reported.

In 2007, interspecies transmission of the entire avian-origin H3N2 was first documented in South Korea^10^. This virus caused clinical signs in dogs ranging from mild respiratory disease to death. During this time, genetically-related H3N2 viruses were also identified in the canine population in several regions of China^11–15^. Subsequent serological surveillance of farm and pet dogs indicated that this virus might have also been circulating in the canine populations of Korea^16^, and that interspecies transmission might have occurred prior to 2007^17^. In experimental settings, this novel CIV demonstrated efficient growth and horizontal transmission in canine and feline hosts, but could not sustain aerosol transmission to ferrets^18–20^. However, the continued circulation of the H3N2 CIV was observed in domestic cats in 2010 in South Korea^21,22^. In addition, natural co-infection with the H1N1 pdm09 virus in a single host resulted in reassortment with the wild type H3N1 virus^23^. Overall, these reports have raised concerns that dogs, the primary human pet, could mediate adaptation of influenza A viruses for zoonotic transmission and thus, might serve as alternative hosts for genetic reassortment of these viruses.

In the present study we compared the genetic and biological properties (e.g., host range and pathogenic potential) of three representative canine H3N2 viruses, A/canine/Korea/AS-01/2009(H3N2) (AS-01/09), A/canine/Korea/AS-05/2012(H3N2) (AS-05/12), and A/canine/Korea/AS-11/2013(H3N2) (AS-11/13), in appropriate avian (chickens and ducks) and mammalian (dogs and ferrets) animal models. Further, we compared each of these viruses with the avian A/duck/Korea/LPM91/2006(H3N2) (LPM91/06) virus, which is the closest genetic ancestor of the avian-origin canine H3N2 viruses in this study^24^. Using recombinant viruses in the backbone of the LPM91/06 virus, we identified the genetic components critical for canine adaptation of avian H3N2 influenza viruses.

## Materials and methods

### Samples and virus isolation

The CIVs used in this study were isolated from nasal swabs and lung tissue samples from dogs that exhibited influenza-like clinical signs at local pet protection shelters and animal hospitals in South Korea from 2009 to 2013 (Table 1). Of these, three viruses, A/canine/Korea/AS-05/2012(AS-05/12), A/canine/Korea/AS-08/2012(AS-08/12) and A/canine/Korea/AS-09/2012(AS-09/12), were isolated from the lungs of dead puppies at three independent pet farms in Chungcheong province. A total of ten canine H3N2 viruses were isolated from canine nasal swab specimens and lung tissues during this study (Table 1). These H3N2 CIVs were isolated from monolayers of Madin-Darby canine kidney (MDCK) cells and were propagated in 11-day-old specific pathogen-free (SPF) embryonated chicken eggs to prepare a virus stock. An avian A/duck/Korea/LPM91/2006(H3N2) (LPM91/06) virus, which is the closest genetic ancestor of the avian-origin canine H3N2 viruses in this study, was isolated during routine surveillance in 2006 from clinically healthy ducks from a live poultry market.

**Table 1:** List of H3N2 viruses isolated from canines in South Korea from 2009 to 2013

Isolation year	Isolation date	Subtype	Virus name	Isolation region	Sample type	Clinical signs	Genetic group
2009	SEP/18	H3N2	A/canine/Korea/AS-01/2009	Dae-Jeon	Nasal swab	Cough, fever, mucopurulent discharge, no mortality	Type I
2012	MAY/10	H3N2	A/canine/Korea/AS-03/2012	Non-San	Nasal swab	Cough, high fever, no mortality	Type III
	JUN/11	H3N2	A/canine/Korea/AS-04/2012	Dae-Jeon	Nasal swab	Cough, high fever, no mortality	Type III
	JUL/14	H3N2	A/canine/Korea/AS-05/2012	Non-San	Lung	Fatal cases (30%) with severe clinical symptoms	Type II
	AUG/24	H3N2	A/canine/Korea/AS-06/2012	Dae-Jeon	Nasal swab	Cough, high fever, no mortality	Type III
	SEP/17	H3N2	A/canine/Korea/AS-08/2012	Non-San	Lung	Fatal cases (28%) with severe clinical symptoms	Type II
	OCT/15	H3N2	A/canine/Korea/AS-09/2012	Non-San	Lung	Fatal cases (25%) with severe clinical symptoms	Type II
2013	JUN/8	H3N2	A/canine/Korea/AS-11/2013	Dae-Jeon	Nasal swab	Sneeze, high fever, no mortality	Type III
	AUG/9	H3N2	A/canine/Korea/AS-14/2013	Non-San	Nasal swab	Cough, high fever, no mortality	Type III
	SEP/11	H3N2	A/canine/Korea/AS-15/2013	Non-San	Nasal swab	Cough, high fever, no mortality	Type III

### Genomic sequencing and phylogenetic analysis

Viral RNA was extracted from infected allantoic fluid using the RNeasy Mini Kit (Qiagen, Valencia, CA, USA) according to the manufacturer’s instructions. Reverse transcription was performed under standard conditions with random hexamer primers, and PCR amplification was performed using universal primers for the influenza A virus^25^. The 50-µL PCR reaction contained 5 U of Enzynomics *n*Taq DNA polymerase (Enzynomics, DaeJeon, Korea), 6 µL of 20 mM Mg^2^, 3 µL of 2.5 mM dNTP, 3 µL of an appropriate concentration of template cDNA, and 1 µL of a 10 pM primer mixture. Each PCR product was amplified under the following conditions: pre-denaturation at 94 °C for 5 min, followed by 35 cycles of denaturation at 94 °C for 30 s, annealing at 56 °C for 30 s, and extension at 72 °C for 1 min 30 s, with a final extension step at 72 °C for 10 min. The viral genes from the gel-purified amplicons were sequenced at Cosmo Genetech (Seoul, South Korea) using an ABI 373 XL DNA sequencer (Applied Biosystems, Foster City, CA). The DNA sequences were edited and aligned using the Lasergene sequence analysis software package (DNASTAR 5.0, Madison, WI). The sequences were aligned using CLUSTAL_V^26^, and phylogenetic trees were constructed using the full-length nucleotide sequences obtained from this study and the sequences available in the GenBank database. The trees were constructed using the HKY model and 40 million steps of MCMC^27^, different models of population dynamics were tested (constant population size, exponential population growth, expansion population growth, logistic population growth, and Bayesian Skyline). The uncorrelated exponential molecular clock was selected according to comparison of Bayes factors with estimates obtained for strict and uncorrelated lognormal local clocks. Results were examined using the TRACER v1.6 program from the BEAST package. Convergence was assessed with ESS (effective sample size) values after a burn-in of 40,000 steps. Models were compared by calculating the Bayes factor (BF) from the posterior output of each of the models using the TRACER v1.6 program as explained on the BEAST website (available at: http://beast.bio.ed.ac.uk/Model_comparison). A log BF (natural log units) >2.3 indicates strong evidence against the null model. Maximum clade credibility trees were generated using Tree Annotator from the BEAST package and FigTree v1.4.3 (available at: http://tree.bio.ed.ac.uk) was used for visualization of the annotated trees.

### Virus titrations

The virus titers of the virus stocks, nasal washes, oropharyngeal and cloacal swabs, and homogenized/clarified organ tissue samples were defined as the 50% egg-infective dose (EID_50_) and were obtained by injecting 200 μL of 10-fold serially diluted samples into 11-day-old SPF embryonated chicken eggs which were harvested 48 h after inoculation. The allantoic fluid was examined with hemagglutination assays using 0.5% chicken red blood cells. The mean virus titers were expressed as the log_10_ EID_50_/mL or log_10_ EID_50_/g of the collected tissue samples^28^. The limit of virus detection was set at <0.7 log_10_ EID_50_/mL or log_10_ EID_50_/g.

### Experimental infection of dogs and ferrets

To examine the pathogenicity and transmissibility of AS-01/09, AS-05/12, AS-11/13, LPM91/06, and each of the reassortant viruses between AS-01/09 and LPM91/06 viruses, influenza antibody-free beagles (two beagles per virus per cage/in duplicate) aged nine to ten weeks were intranasally inoculated with 10^5.5^ EID_50_ of the virus in a volume of 1 mL (500 µL per nostril). The next day, two naïve dogs were introduced into the adjacent cage, spatially separated from the infected dogs by two stainless steel grids 5 cm apart. Nasal secretion swabs were collected from infected dogs at 1, 3, 5, and 7 days post infection (dpi) and from contact dogs daily until 9 days post contact (dpc). Rectal temperatures were measured with a digital predictive thermometer with the use of lubricant. The rectal digital thermometer (RDT) was inserted into the rectum for a length of about 2 cm. Time of a single rectal measurement for each dog was 10–20 s. In between dogs, RDT was disinfected with 70% ethanol. Five days after infection, two inoculated dogs from each group were euthanized for virus titration in the trachea and lungs. Sera were collected at 14 and 21 dpi, and seroconversion was analyzed using a hemagglutination inhibition (HI) assay. To rule out other secondary *Mycoplasma* species infection during the study, we tested all the collected lung tissues for species-specific PCR reactions for *M. arginini, M. canis, M. cynos, M. edwardii, M. felis, M. gateae, M. maculosum, M. molare, M. opalescens, M. spumans, Mycoplasma sp. HRC 689*, and *M. collis* as previously described^29^. Further, Canine parvovirus, Canine distemper virus, and seasonal H1N1 and H3N2 influenza A viruses were tested for any opportunistic infection by (RT)-PCR with each specific primer set. To assess the transmissibility of the three viruses in ferrets, groups of ferrets (*n* = 4) (*Mustela putorius furo*) (Marshall Bio Resources, NY, USA) seronegative for influenza virus antibody were intranasally inoculated with 10^7.0^ EID_50_ of each H3N2 virus in a volume of 1 mL (500 µL per nostril). The next day, two naïve ferrets were introduced into the adjacent cage and spatially separated from infected ferrets by two stainless steel grids, 5 cm apart. Five days after infection, two of the infected ferrets in each group were euthanized for viral titration in the brain, trachea, lung, kidney, spleen, and intestines (rectum). Sera were collected 14 and 21 dpi, and seroconversion was analyzed using a HI assay.

### Growth kinetics of virus in vitro

To establish the multistep growth curves for each virus, MDCK and differentiated primary normal human bronchial epithelial (NHBE) cells were infected in triplicate in six-well plates with AS-01, AS-05, AS-11, and LPM91 at multiplicities of infection of 0.001 or 0.01, respectively. After incubation at 35 °C for 1 h, the viral inoculates were replaced with serum-free medium containing L-1-tosylamido-2-phenylethyl chloromethyl ketone (TPCK)-treated trypsin (0.3 μg/mL) (Sigma-Aldrich) appropriate for each cell line. Cell culture supernatants were harvested at 12, 24, 48, and 72 h post infection (hpi) for virus titration in MDCK cells, as described above. Viral growth rates in all cells were determined at three times in duplicate at 35 °C. The virus titers were determined as log_10_ TCID_50_/mL in MDCK cells.

### Experimental infection of chickens and ducks

The same experimental set up was used to assess the pathogenicity and transmissibility of the three viruses in avian species. A total of 12 six-week old SPF, White Leghorn chickens (*G. gallus domesticus*) and influenza-seronegative domestic ducks (*Anas platyrhynchos domesticus*) were intranasally inoculated with 10^5.5^ EID_50_ of each virus in a volume of 1 mL (four birds per virus per cage). Two naïve counterparts were introduced into the same cage 1 dpi. The birds were observed daily for clinical signs of disease. Tracheal and cloacal swabs were collected every other day for 7 days from the infected group and every day for 9 days from the contact groups. Viral titers in the lung and trachea were also examined after euthanizing two birds from each group at 5 dpi. Serum samples were collected from all birds 21 dpi or dpc and were subsequently tested using an HI assay.

### Plasmids and generation of recombinant viruses

All eight gene segments of the avian LPM91/06 virus and the canine virus AS-01/09 were cloned into the pHW2000 plasmid vector to generate reverse genetics (RG) viruses, as previously described^30^. The LPM91-Ca-PB2, LPM91-Ca-PB1, LPM91-Ca-PA, LPM91-Ca-HA, LPM91-Ca-NA, LPM91-Ca-NP, LPM91-Ca-M, LPM91-Ca-NS, and LPM91-Ca-NA/NP/M recombinant viruses were rescued in 6-well plates of co-cultured 293T HEK and MDCK cells (3:1 ratio) transfected with the eight viral plasmids as indicated, each containing 1 µg of the respective gene segment. The cells were transfected using the TransIT-LT1 transfection reagent (Mirus Bio) according to the manufacturer’s instructions. The transfection medium was removed after 6 h and replaced with Opti-MEM I (Gibco) containing 0.3% bovine serum albumin and 0.01% FBS. After 30 h, 1 mL of Opti-MEM I containing 0.2 µg/mL of L-1-tosylamido-2-phenylethyl chloromethyl ketone (TPCK)-treated trypsin (Sigma-Aldrich) was added to the transfected cells. The supernatant was harvested after 48 h and injected into 11-day-old embryonated chicken eggs to propagate the virus. All rescued viruses were re-sequenced to ensure the absence of unwanted mutations and the presence of the desired gene combinations (Cosmo Gene Tech; Seoul, Republic of Korea). The infectivity of the stock viruses was determined after calculating the EID_50_ according to the method of Reed and Muench^28^.

### Histopathology

Lung tissue was isolated from dogs 5 days after inoculation. To prevent cross-contamination, different sterile instruments were used to collect each tissue sample. The collected tissues were fixed in 10% neutral-buffered formalin. Immunohistochemistry was performed to examine the distribution of CIV antigens in the lungs and other organs of infected animals using a homologous canine polyclonal antibody against the AS-01/09 virus.

### Ethics statement

All animal experiment performed followed general animal care guidelines mandated under the Guidelines for Animal Use and Care of the Korea Center for Disease Control (K-CDC). They were approved by the Medical Research Institute and Laboratory Animal Research Center (LARC) (approval number: CBNUA-957-16-01), a member of the IACUC of Chungbuk National University in Korea. Animal experiments were undertaken in partnership with Bioleaders Corp. (permit number: BLS-ABSL-10-003).

### Statistical analysis

The differences among log_10_-transformed viral titers of different viruses were compared using one-way ANOVA analysis of variance. Statistical analyses were carried out using GraphPad Prism^TM^ software (v5) (La Jolla, CA, USA).

## Results

### Phylogenetic and genomic sequence analysis

To identify the genetic origins of the CIV H3N2 isolates obtained between 2009 and 2013, the full-length sequences of all eight segments of each virus were compared with those of other influenza viruses isolated from different hosts (i.e., avian and equine species). Each viral segment demonstrated high nucleotide sequence homology (≥97–99%) with other H3N2 CIVs isolated in Korea and China since 2007, which were closely related to avian viruses circulating in the live poultry markets of Korea (surface genes) and wild birds (remaining segments)^10^. Specifically, the phylogenetic analysis revealed that all gene segments diverged from North American, avian-lineage viruses, but clustered together with 2007 and 2010 Korean H3N2 CIVs from dogs and cats, respectively (Supplementary Figure S1). No evidence of further viral reassortment was observed.

**Fig. 1:**
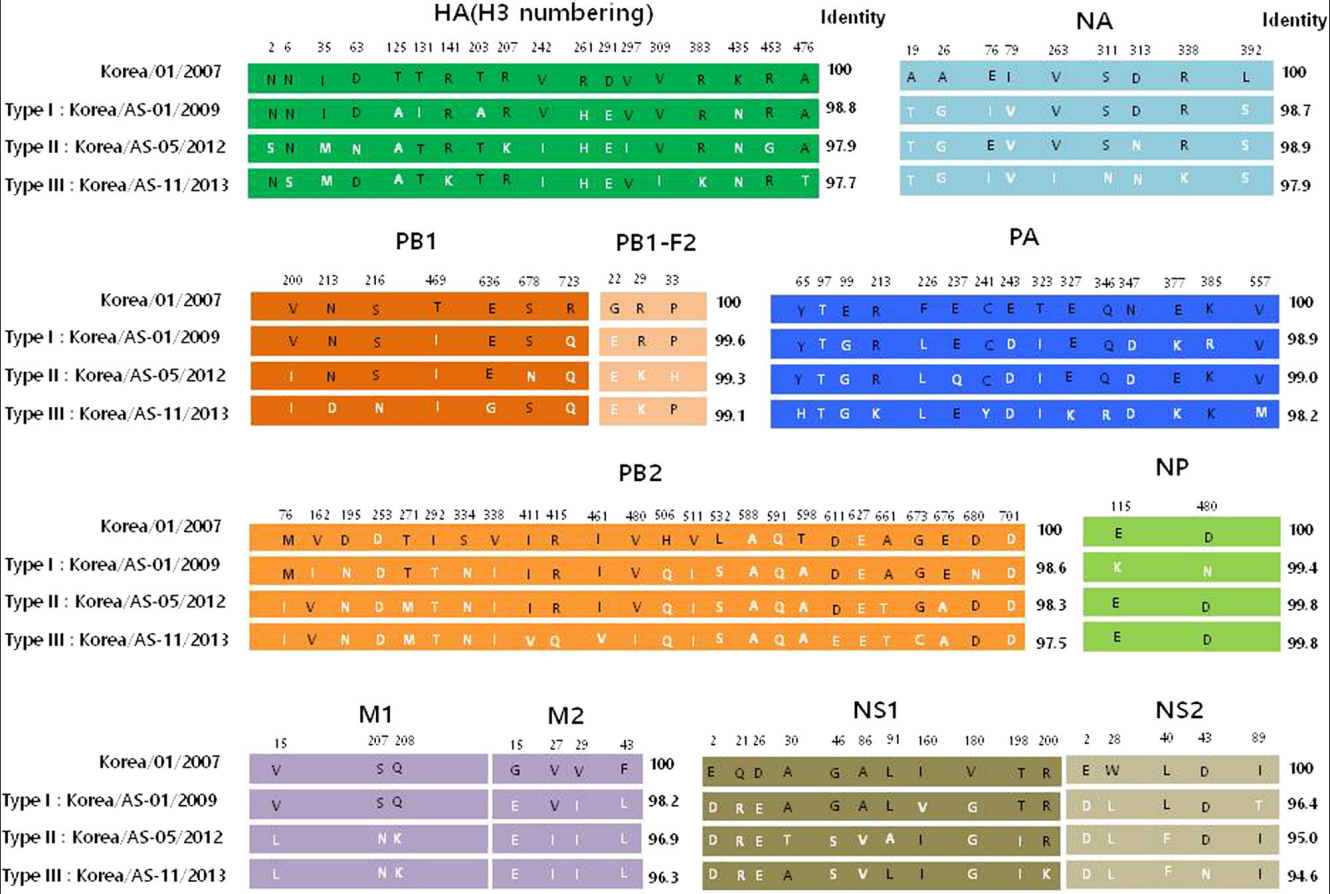
Schematic diagram of commonly divergent amino acids of each gene segment in CIVs from 2007 to 2013 canine isolates. Differences in the amino acid residues of each gene are indicated by single-letter amino acid codes with positions indicated at the top of the diagram. The black and white letters indicate the amino acid regions originating from the first Korean CIV and recent CIVs, respectively. The amino acid positions of HA are based on the numbering of the H3 HA. Type I includes only one A/canine/Korea/AS-01/2009 virus. Three individual isolates (AS-05/12, AS-08/12, and AS-09/12 viruses) comprise Type II viruses. Type III viruses were the most divergent viruses from the Type I virus, containing six individual isolates (AS-03/12, AS-04/12, AS-06/12, AS-11/13, AS-14/13, and AS-15/13 viruses)

The mature HA proteins of all Korean CIVs contain glutamine (Q) at position 226 and glycine (G) at position 228 (H3 numbering), conferring binding to avian-type receptors^31^, whereas the HA cleavage sites bear PERQTR/G sequence motifs (Table 2). For comparison, the North American equine and canine H3 viruses contain the PEKQTR/G or PEKQIR/G motif. All Korean CIVs tested in this study also share six glycosylation sites with previous Korean and Chinese isolates on HA proteins at positions 22, 38, 81, 165, 285, and 483. Notably, the AS-05/12, AS-08/12, and AS-09/12 (Type II viruses) contain an aspartic acid (D) to asparagine (N) substitution at position 63 (the antigenic site, E1) resulting in an additional glycosylation site. Further, the AS-05/12, AS-08/12, and AS-09/12 (Type II viruses) contain a lysine (K) residue at position 207 (the antigenic site D), which was similarly patterned to North American equine and canine viruses (Table 2). For comparison, the North American equine and canine H3 viruses shared five N-glycosylation sites with each other (8, 22, 38, 53, 165, and 285 on HA proteins), while the A/equine/Kentucky/5/2002 (H3N8) virus has an additional site at residue 483 which is commonly found in Asian CIV and avian H3N2 viruses (Table 2). All Korean CIVs harbored a three-amino-acid deletion in the stalk region of the NA genes compared to A/canine/Jiangsu/1/2009 (H3N2). In addition, type-specific mutations were observed in the remaining viral protein segments of CIVs. However, none of these mutations were associated with previously known molecular markers for transmissibility, virulence or drug resistance, except for a valine (V)-to-isoleucine (I) M2 substitution (V27I) from AS-05/12 to AS-11/13, which might confer amantadine-resistance (Fig. 1). Taken together, phylogenetic and genomic analysis revealed that Korean H3N2 CIVs undergo continuous genetic evolution in the dog population and at least three types of CIVs can be differentiated in the present study based on the amino acid substitution pattern: Type I (AS-01/09) was considered an old strain of recent Korean CIVs, Type II (AS-05/12) was the virus strain isolated from the lungs of dead dogs, and Type III (AS-11/13) was the most recently identified CIV that had mutated from Type-II CIVs (Fig. 1). Therefore, we selected a representative strain of each type (AS-01/09, AS-05/12, AS-11/13) for more detailed analysis.

**Table 2:** Amino acid sequence comparison between virus isolates

HA (H3 numbering)															NA
Virus	Antigenic sites									Cleavage site	Receptor binding sites			N-glycosylation sites	Stalk region deletion
	A			B	C		D		E						
	121-126	131-137	140-146	155-163	53- 54	275- 278	200-215	62- 63	78-83	324-330	222	226	228	Asn-Xaa-Ser/Thr (N-X-S/T)	74-79 (EKEKEI)
A/canine/Jiangsu/01/2009/ H3N2	ITEGFT	TQNGGSG	KRGPANG	TKSGNTYPV	NN	DTCI	GRVTSTRRSQQTIIP	RD	VFQNET	PERQTRG	L	Q	G	22, 38, 81, 165, 285, 483	NO (EKEKEI)
A/canine/Korea/01/2007/ H3N2	ITEGFT	TQNGGSG	KRGPANG	TKSGNTYPV	NN	DTCI	GRVTVSTRRQQTIIP	RD	VFQNET	PEKQTRG	L	Q	G	22, 38, 81, 165, 285, 483	YES (EK---I)
A/canine/Korea/AS-01/2009/ H3N2 (Type I)	ITEGFT	IQNGGSG	KRGPANG	TKSGNTYPV	NN	DTCI	GRVAVSTRRSQQTIIP	RD	VFQNET	PERQTRG	L	Q	G	22, 38, 81, 165, 285, 483	YES (EK---I)
A/canine/Korea/AS-05/2012/ H3N2 (Type II)	ITEGFT	TQNGGSG	KRGPANG	TKSGNTYPV	NN	DTCI	GRVTVSTKRSQQTIIP	RN	VFQNET	PERQTRG	L	Q	G	22, 38, 63, 81, 165, 285, 483	YES (EK---I)
A/canine/Korea/AS-11/2013/ H3N2 (Type III)	ITEGFT	TQNGGSG	KKGPANG	TKSGNTYPV	NN	DTCI	GRVTVSTRRSQQTIIP	RD	VFQNET	PERQTRG	L	Q	G	22, 38, 81, 165, 285, 483	YES (EK---I)
A/canine/Florida/43/2004/ H3N8	TAEGFT	TQNGRSG	KRGSADS	TKSGSSYPT	NK	DICV	GRVTVSTKRSQQTIIP	RN	AFQYES	PEKQTRG	L	Q	G	8, 22, 38, 53, 165, 285	(IERPSN)
A/equine/Kentucky/5/2002/ H3N8	TAEGFT	TQNGRSG	KRGSADS	TKSGNSYPT	NK	DICV	GRVTVSTKRSQQTIIP	RN	VFQYEN	PEKQIRG	W	Q	G	8, 22, 38, 53, 165, 285, 483	(IERPPN)
A/duck/Korea/LPM91/2006/ H3N2	ITEGFT	TQNGGSG	KRGPANG	TKSGNAYPV	SN	DTCI	GRVTVSTRRSQQTIIP	RD	VFQDET	PEKQTRG	W	Q	G	8, 22, 38, 165, 285, 483	YES (EK---I)

### Pathogenicity and transmissibility in dogs

**Fig. 2:**
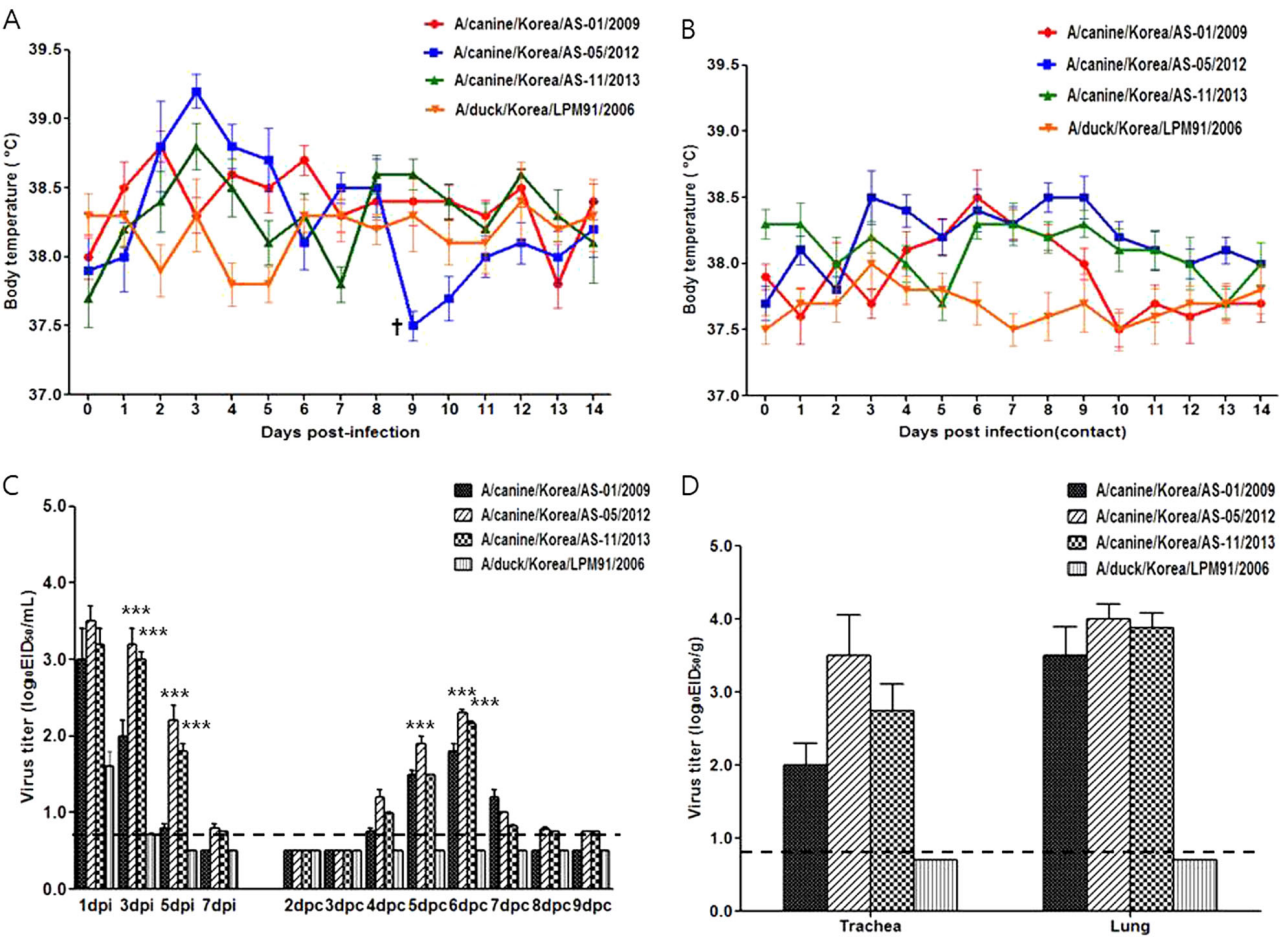
Pathogenicity and transmissibility of AS-01/09, AS-05/12, AS-11/13, and LPM91/2006 viruses in beagles. The body temperatures of infected (**a**) and contact (**b**) beagles (†succumbed to death) were monitored. Groups of four beagles inoculated with 10^5.5^ EID50 of each virus were individually placed adjacent to naïve (contact) beagles. The mean viral titers (log_10_ EID_50_/mL) for nasal-wash (**c**) or lung and tracheal (**d**) samples are shown for each group of beagles. The limit of virus detection was 0.7 log_10_ EID_50_/mL or gram. Statistically significant differences (*P < 0.05, **P < 0.001, and ***P < 0.0001) between each representative canine and AS-01/09 viruses are indicated by *P value was determined using one-way ANOVA

Because each representative virus was isolated from different clinical cases of canine infections (Table 1), we compared the pathogenic potential of these viruses as well as the closest genetic ancestor of the avian-origin canine H3N2 viruses in this study, A/duck/Korea/LPM91/2006 (LPM91/06)^24, 32^, via experimental intranasal infection with 10^5.5^ EID_50_/mL of each virus in seronegative beagles. Substantially elevated body temperatures were observed 2–3 dpi (>39.0 °C) in beagles inoculated with the three canine viruses, but not with the avian virus (Fig. 2a). In addition, indirect respiratory droplet (RD)-contact dogs of H3N2 CIV isolates showed slightly increased body temperatures (<38.5 °C) with mild cough and mucopurulent discharge from 6 to 8 dpi, which normalized thereafter (Fig. 2b). By contrast, neither the infected nor the RD-contact group of LPM91/06 showed elevated body temperatures or any clinical signs (sneezing or mucopurulent discharge) of infection (Fig. 2a, b, and data not shown). However, all AS-01/09, AS-05/12, and AS-11/13-infected groups exhibited mucopurulent discharge, mild cough, and lethargy at 2 dpi, which persisted until 4 dpi. Notably, one of the beagles inoculated with the AS-05/12 virus showed a loss of appetite and exhibited fatigue, with markedly reduced physical activity at 9 dpi and eventual death. Mild cough was only observed in the RD-contact groups of the AS-01/09 and AS-11/13 CIVs beginning at 4 dpc and persisting until 8 dpc, whereas the AS-05/12 RD-contact group showed higher body temperatures that persisted until 9 dpc (Fig. 2b). To rule out other secondary Mycoplasma species infections, we tested all collected samples for various canine pathogens such as Mycoplasma species, Canine parvovirus, Canine distemper virus, and seasonal influenza A viruses by (RT)-PCR with each specific primer set. However, no pathogens were detected other than AS-05/12 CIV (data not shown).

All infected viruses were detected in nasal swabs obtained from inoculated dogs at 1 dpi (CIVs = 3.0–3.5 log_10_ EID_50_/mL and AIV = 1.5 log_10_ EID_50_/mL) (Fig. 2c). While LPM91/06 could not sustain viral growth beyond 1 dpi, the three CIVs persisted 5–7 dpi. It is possible that LPM91/06 cannot infect dogs, and the virus detected at 1 dpi may be remnant in the nasal cavity from the virus inoculation. The nasal swab titers of AS-05/12 and AS-11/13 were at least 10 times higher than that of AS-01/09 at 3 and 5 dpi (*P* < 0.0001). All RD-contact dogs for the three CIV-infected groups were positive for virus transmission at 4 dpc, with peak titers of 2.0–2.5 log_10_ EID_50_/mL at 6 dpc (Fig. 2c). By contrast, the avian-derived LPM91/2006 virus was not recovered from any of the RD-contact dogs.

Virus titration in trachea and lung tissue samples harvested at 5 dpi from two of the inoculated dogs from each group showed that AS-05/12 produced titers at least 10 times higher than that of AS-01/09 in the trachea (3.5 vs. 2.0 log_10_ EID_50_/g), although comparable titers were observed with that of AS-01/09 in lungs (4.0 vs. 3.5 log_10_ EID_50_/g) (Fig. 2d). LPM91/06 was not detected in any of the tissue samples tested (<0.7 log_10_ EID_50_/g detection limit).

### In vitro analysis of CIV growth properties

To further examine the growth properties of the representative CIVs (AS-01/09, AS-05/12, and AS-11/13) and their closet genetic ancestor, avian influenza virus (LPM91/06), in vitro, the viral growth kinetics of each was examined in MDCK and human bronchial epithelial (NHBE) cells (Supplementary Figure S2). In MDCK cells, the replication of two CIVs (AS-01/09 and AS-05/12) and avian influenza virus (LPM91/06) were markedly higher at 48 hpi (peak titers of 6.1–7.5 log_10_TCID_50_/mL) compared to the AS-11/13 virus. However, AS-11/13 showed low viral titers (peak titers of 2.8 log_10_/TCID_50_/mL) compared with both the other CIVs (AS-01/09 and AS-05/11) and avian influenza virus (LPM91/06) (Supplementary Figure S2). In primary NHBE cells AS-01/09, AS-05/12, and LPM91/06 reached comparable titers at 48–72 h (peak titers of 4.8–5.5 log_10_ TCID_50_/mL) while AS-11/13 showed significantly lower viral titers (peak titers of 2.8 log_10_ TCID_50_/mL) (Supplementary Figure S2).

### Pathogenicity and transmissibility in ferrets

**Fig. 3:**
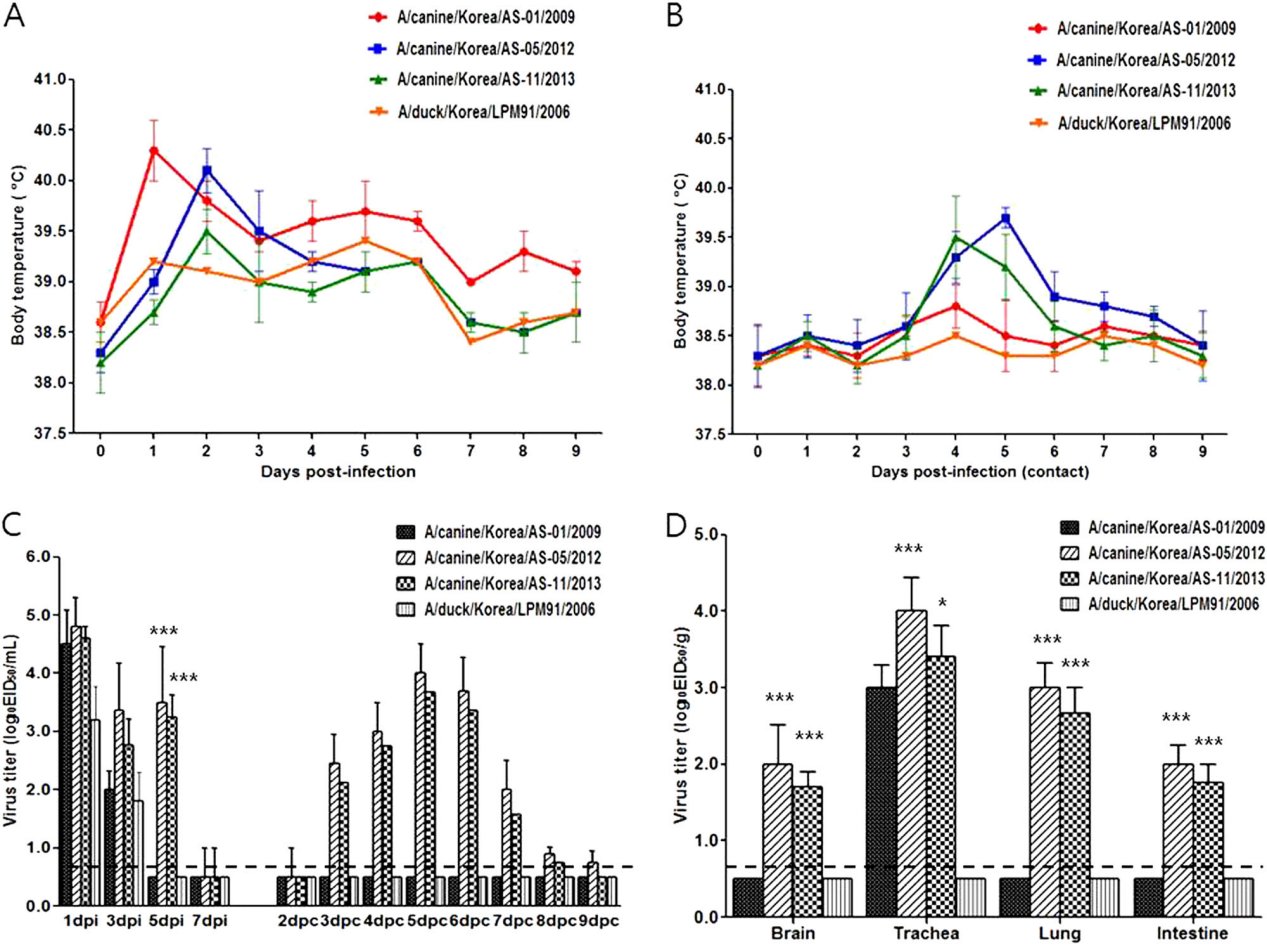
In vitro characterization of each representative type of canine influenza virus (AS-01/09, AS-05/12, and AS-11/13) and their ancestor virus (LPM91/06) in mammalian cell lines. Each virus was infected at an MOI of 0.001 in MDCK cells (**a**) or 0.01 in NHBE cells (**b**) in the presence of L-1-tosylamido-2-phenylethyl chloromethyl ketone (TPCK)-treated trypsin. Supernatants of virus cultured cell lines were harvested at 12, 24, 48, and 72 hpi, and virus titers were measured in MDCK cells. The virus titers were determined as means ± SD from three independently performed experiments. Dotted lines indicate the limit of detection (1.8log_10_TCID_50_/mL). Statistically significant differences (*P < 0.05, **P < 0.001, and ***P < 0.0001) between each representative canine and AS-01/09 viruses are indicated by *P value was determined using one-way ANOVA

Because dogs are one of the most common human companion animals, CIVs pose a significant threat to public health. To evaluate the pathogenic potential of the H3N2 viruses in a surrogate animal model for humans, groups of antibody-free ferrets (*n* = 4) were intranasally infected with 10^7.0^ EID_50_ of the AS-01/09, AS-05/12, AS-11/13, or LPM91/06 viruses. Experimental inoculation with the CIV strains increased ferret body temperature on 1 and 2 dpi, whereas LPM91/06 virus infection did not result in significant changes in body temperature (Fig. 3a). Furthermore, the AS-05/12 (2/2) and AS-11/13(1/2) canine isolates were detected among RD-contact ferrets as early as 3 dpc and infection was accompanied by increases in body temperature suggestive of fever (Fig. 3b, c). Although all viruses replicated in the upper nasal cavity, the canine AS-05/12 and AS-11/13 viruses consistently produced higher nasal wash titers than the LPM91/06 or AS-01/09 viruses and were persistently detected up to 5 dpi (Fig. 3c). All infected ferrets exhibited mild to frequent sneezing from 2 dpi which persisted until 3 dpi, however no clinical signs of severe infection, such as weight loss or anorexia, were observed (data not shown).

**Fig. 4:**
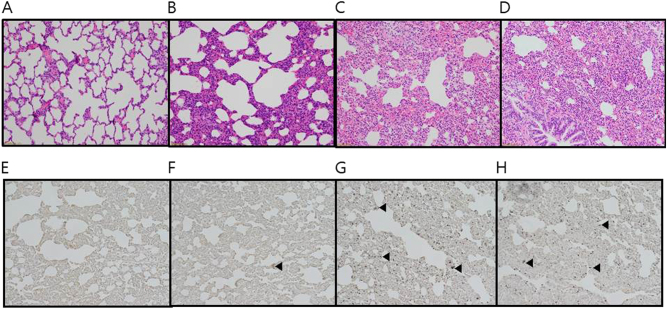
Histopathology (a–d) and immunohistochemistry with anti-influenza NP antigen antibody (e–h) of lung samples obtained from ferrets infected with each H3N2 influenza isolate. Ferrets were intranasally inoculated with 10^7.0^ EID_50_/mL of each virus. Lungs were harvested 5 days after virus inoculation. LPM91/06 (**a** and **e**), AS-01/09 (**b** and **f**), AS-05/12 (**c** and **g**) and AS-11/13 (**d** and **h**), respectively. No damage was evident in lung sections from the LPM91/06-infected group (**a** and **e**). Moderate to severe lung damage, indicated by lesions, are shown in the AS-01/09 (**b** and **f**), AS-05/12 (**c** and **g**) and AS-11/13 (**d** and **h**) groups. Arrows indicate positive immunostaining

All three canine H3N2 viruses were recovered from the tracheal tissue samples obtained from infected ferrets euthanized at 5 dpi (Fig. 3d). However, AS-05/12 and AS-11/13 were also detected in the brain, lung, and intestinal tissue samples (Fig. 3d). Virus was not detected in any samples obtained from LPM91/06-infected ferrets. Histologically, all those slides of lungs (LPM91/06, AS-01/09, AS-05/12, and LPM91/06) have lesions with different severity (Fig. 4). These slides commonly consist of varying sized compressed areas in which are patches of thickened alveolar septa with increased number of interstitial cells such as lymphocytes, macrophages and occasionally plasma cells interpreted to be interstitial pneumonia. There is free of exudates in the lumen alveolar space and bronchioles. The histologic lesions of LPM91/06 or AS-01/09-infected lungs showed limited or moderated compressed areas respectively (Fig. 4a, b). However, the histologic lesions of AS-05/12 and AS-11/13-infected lungs showed more compressed areas compared to those of LPM91/06-infected lung (Fig. 4a, c, d). Accordingly, moderate to severe histopathology was only observed in the lungs harvested from AS-05/12- and AS-11/13-infected ferrets, where pulmonary viral growth was confirmed via immunohistochemistry (IHC) with anti-influenza NP antigen antibody (Fig. 4e-h). Serum antibody titers, measured by HI assay, were detected at 14 dpi among ferrets infected with the three canine viruses. Furthermore seroconversion (at 40 HI titers) was observed among the RD-contacts of the AS-05/12 (2/2)- and AS-11/13 (1/2)-infected groups (data not shown).

### Pathogenicity and transmissibility in terrestrial poultry

Because this virus is of avian origin, pathogenic assessment was also extended to domestic poultry species. Groups of ducks and chickens were intranasally inoculated with 10^5.5^ EID_50_ of each virus in a volume of 1 mL and then were co-housed one day later with naïve contact birds. None of the three canine H3N2 isolates could efficiently replicate in either avian species (Supplementary Figure S3). Consequently, none of the H3N2 CIVs was transmitted to contact birds via the oral-to-fecal route, as demonstrated by the lack of detection or seroconversion at 21 dpc (data not shown). By contrast, the avian H3N2 LPM91/06 virus progressively replicated in ducks, reaching peak titers of 10^3.0^ EID_50_/mL in tracheal swabs persisting up to 5 dpi, and this virus was also detected in the cloacal swabs from 3 to 5 dpi (Supplementary Figure S3). In addition, LPM91/06 was readily transmitted to co-housed ducks (Supplementary Figure S3). However, in a manner similar to that of H3N2 CIVs, it did not successfully infect inoculated chickens (Supplementary Figure S3). This lack of infection was further supported by the absence of antibody titers in the sera of inoculated and contact chickens at the end of the experimental period (21 days).

### The roles of individual CIV gene segments for canine adaptation

Although the Korea CIV appears to share a common genetic ancestor with avian H3N2 viruses (Supplementary Figure S1), animal infection studies showed that each virus could only infect a specific host. Therefore, to identify the canine-specific adaption factor in CIVs, we generated reassortment viruses by placing each gene segment of the canine AS-01/09 virus, the earliest canine-adapted virus used in the present study, in the avian LPM91/06 virus background (Table 3). Additionally, we added the LPM91-Ca-NA/NP/M virus, which was the only rescued virus from the co-infection of canine AS-01/09 and LPM91/06 viruses. The rescued reassortant viruses replicated relatively well in 11-day-old embryonated chicken eggs infected with 10^6.5–8.5^ EID_50_/mL (Table 3).

**Table 3:** List of canine H3N2 RG viruses in the backbone of the LPM91/06 virus

Virus	Virus titer (EID_50_/mL)	Canine transmission	Seroconversion in RD contact dogs (HI)^a^
LPM91-Ca-PB2	8.5	0/4	10>
LPM91-Ca-PB1	7.8	0/4	10>
LPM91-Ca-PA	6.5	0/4	10>
LPM91-Ca-HA	8.3	0/4	10>
LPM91-Ca-NA	7.0	3/4	40
LPM91-Ca-NP	6.7	3/4	40
LPM91-Ca-M	7.5	4/4	40
LPM91-Ca-NS	7.5	0/4	10>
LPM91-Ca-NA/NP/M	7.5	4/4	80

^a^ HI titer of sera obtained from contact dogs after 21 days of contact

**Fig. 5:**
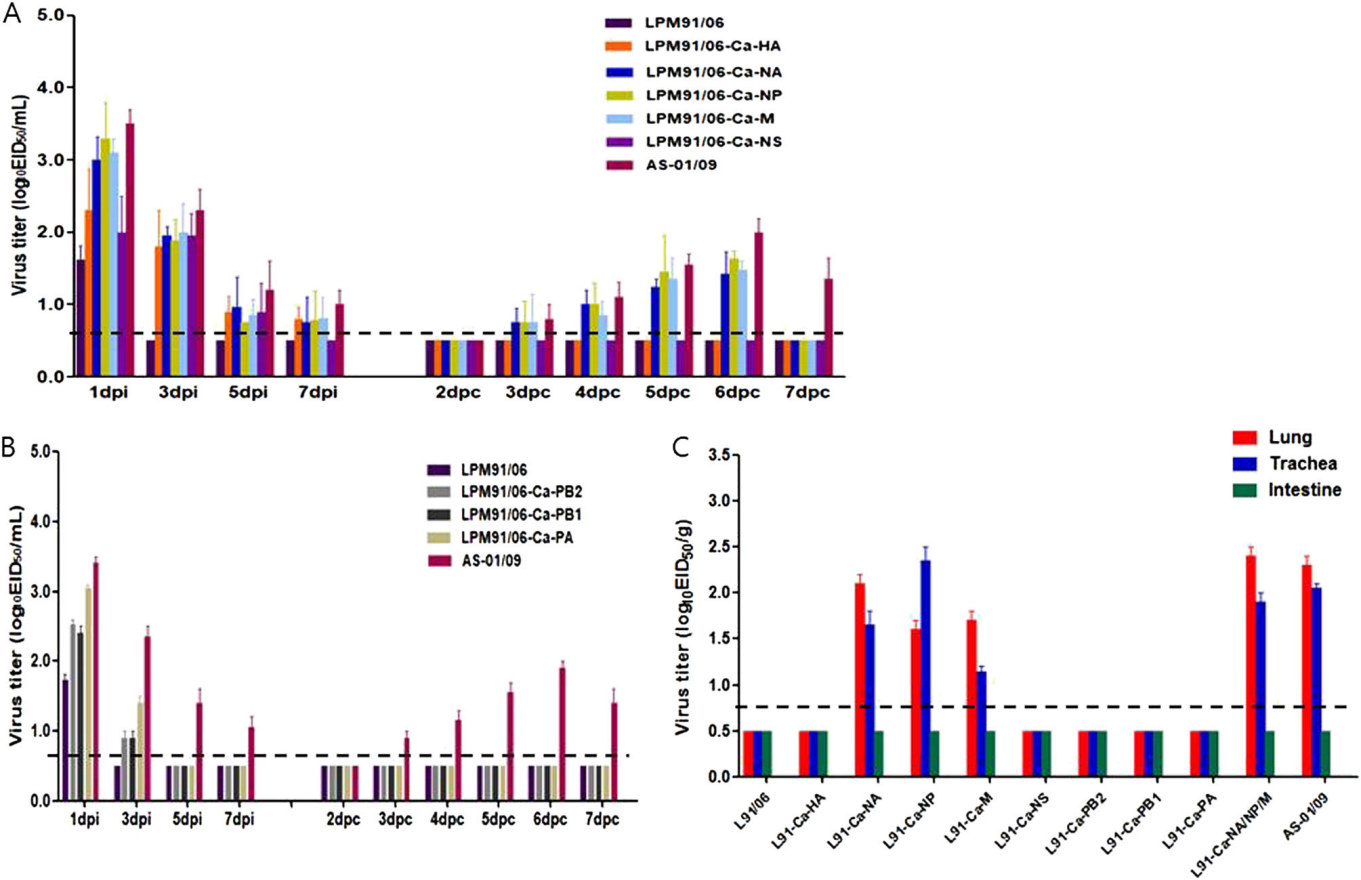
Replication and transmission of reassorted viruses in dogs. Groups of four dogs were inoculated with 10^5.5^ TCID_50_/mL of each reassorted virus (Table 3). RD-contacts were individually placed adjacent to the inoculated dogs at 1 dpi. Nasal wash titers are shown for individual dogs (**a** and **b**). Two dogs per group were humanely euthanized at 5 dpi for virus titration in various tissues (**c**). Virus titers in nasal washes and homogenized tissues are expressed as log_10_EID_50_/mL or gram of tissue collected, with the limit of virus detection set at 0.7 log_10_ EID_50_/mL or gram

Each group of naïve dogs (*n* = 4) was intranasally inoculated with 10^5.5^ EID50/mL of each gene-swapped virus and naïve RD-contact dogs (*n* = 2) were introduced to the adjacent cage one day later, as described in the Materials and Methods. Experimental inoculation with the RG viruses resulted in elevated body temperatures 1 and 2 dpi, whereas no remarkable changes were observed in the LPM91-Ca-NS virus-inoculated groups (data not shown). Although the LPM91/06 virus was not detected in the nasal washes at 3 dpi, the LPM91-Ca-HA, LPM91-Ca-NA, LPM91-Ca-NP, LPM91-Ca-M, and LPM91-Ca-NA/NP/M viruses consistently produced nasal wash titers and were persistently detected up to 7 dpi (Fig. 5a, b). Furthermore, the LPM91-Ca-NA, LPM91-Ca-NP, and LPM91-Ca-M viruses were recovered from RD-contact dogs as early as 3 dpc (Fig. 5a and Table 3). However, the viral titers were relatively low (peaked at 5 or 6 dpc with 10^1.5–1.8^ EID_50_/mL) and disappeared early (by 7 dpc) compared with the wild-type AS-01/09 virus, which persisted up to 7 dpc (peaked at 6 dpc with 10^2.0^ EID_50_/mL) (Fig. 5a). Interestingly, among the reassortant virus infected dogs, the LPM91-Ca-NA/NP/M virus showed the highest viral titer in nasal washes and was readily transmitted into RD-contact animals at 3 dpc with a peak viral titer of 10^1.8^ EID_50_/mL at 6 dpc that persisted up to 7 dpc. This pattern was similar to that of the wild-type AS-01/09 virus (Fig. 5a). Canine polymerase substituted reassortants (LPM91-Ca-PB2, LPM91-Ca-PB1, and LPM91-Ca-PA viruses) could not replicate in nasal cavities of infected dogs compared with other reassortant viruses. Moreover, none of the canine polymerase reassortant viruses were detected at 5 dpi and no virus was transmitted into contact dogs (Fig. 5b).

To investigate the viral replication properties in each organ, two dogs from each virus-infected group were sacrificed at 5 dpi. Consistent with the detection of low viral titers in nasal washes, the LPM91/06, LPM91-Ca-HA, LPM91-Ca-NS, LPM91-Ca-PB2, LPM91-Ca-PB1, and LPM91-Ca-PA viruses were not detected in lungs, trachea, or intestine from infected dogs, while in comparison the LPM91-Ca-NA, LPM91-Ca-NP, and LPM91-Ca-M viruses were recovered from lungs and trachea of infected dogs at 5 dpi (Fig. 5c). Further, comparable virus titers were observed in tissues of RG LPM91-Ca-NA/NP/M virus infected dogs as in wild-type AS-01/09 virus infected animals (Fig. 5c). Thus, these results suggest that a combination of the NA, NP, and M gene segments of canine origin are critical for the adaptation of avian H3N2 viruses in canine hosts, and these gene combinations are enough to support viral growth with persistent infection and more importantly, dog-to-dog transmission of the avian H3N2 virus.

## Discussion

One distinguishing feature of influenza A viruses is their broad host range. Adaptation drives this feature by promoting host specificity and restricting interspecies transmission^4^. In the present study, we report the isolation of H3N2 CIVs genetically related to strains identified in dog populations in South Korea and China since 2007. Further, molecular analyses revealed that these 2012 and 2013 CIVs clearly evolved from the original CIV strains of South Korea and China, including the AS-01/09 virus (Fig. 1). Notably, AS-01/09 (Type I) as well as AS-11/13, AS-03/12, AS-04/12, AS-06/12, AS-14/13, and AS-15/13 viruses (Type III) was isolated from dogs that showed moderate to severe respiratory disease but eventually recovered. However, the AS-05/12, AS-08/12, and AS-09/12 viruses (Type II) were all recovered post-mortem from the lungs of animals obtained from three different canine pet protection shelters. Further investigation revealed that the dogs suffered from severe respiratory disease along with other canines at the same shelter, with an overall mortality rate of approximately 22–55% (personal communication) (Table 1). Although the virus isolation timeline and sources are different, the accumulation of gradual mutations from 2012 to 2013 throughout all eight genes was observed in these viruses (Supplementary Figure S1 and Fig. 1). For example, these include the V27I residue substitution in the M2 protein, which might confer amantadine-resistance (Fig. 1). Thus, these findings suggest the continuous evolution of Korean CIVs rather than the introduction of a new gene pool.

As stated, some H3N2 CIVs cause severe respiratory disease and mortality in dogs^10,15^. In the beagle infection experiments, all three CIVs replicated well compared with an H3N2 avian strain (LPM91/06) was transmitted via aerosol to contact dogs. Notably, AS-05/12 replicated at high titers in the upper and lower respiratory tracts and induced more severe clinical disease symptoms than AS-01/09, eventually resulting in mortality. Conversely, these H3N2 CIVs could not efficiently infect and propagate in domestic avian species compared to LPM91/06, which is consistent with the results seen for a genetically-related Chinese isolate^15^. These data indicate that the canine H3N2 viruses in Asia have undergone significant genetic adaptation from avian to canine species. Therefore, the establishment of these viruses among canine populations is not surprising. Experimental studies of the 2007^18^ and 2008^19^ Korean H3N2 CIV isolates revealed that these viruses could replicate without pre-adaptation but did not spread via indirect or aerosol transmission in ferrets, a widely-accepted animal model used to study the transmissibility and pathogenesis of influenza viruses. Although AS-01/09, AS-05/12, and AS-11/13 all demonstrated viral replication in this host, AS-05/12 consistently showed higher titers in the upper nasal cavity. In addition to the respiratory organs, this virus was also detected in extra pulmonary tissues, such as brain and intestines. Although more detailed studies are needed due to a lack of known virulence markers in mammalian hosts, similar ferret brain infections were reportedly caused by CIVs when a high dose of virus was administered nasally^19,33,34^. Further, there are reports that some influenza viruses can be detected in the intestinal tracts of infected mammalian hosts^33,35,36^. Specifically, the 2009 pandemic H1N1 virus, which could not induce systemic infections in ferrets, was detected in the intestines of infected hosts^37^. Furthermore, the AS-05/12 and AS-11/13 isolates were successfully and indirectly transmitted to naïve ferrets via respiratory airborne droplets, suggesting that these recently-identified H3N2 CIVs might be readily transmitted among mammalian hosts. However, it should be noted that CIV infections may differ from human influenza infections in many ways, including method of viral transmission, viral dissemination, clinical features, pathogenesis, and host immune response even though ferret is considered suitable animal model for influenza virus transmission experiments. Although human cases have not yet been reported, because dogs are companion animals in many human households the continued circulation of these H3N2 CIVs is of great concern due to the possibility of future zoonotic transmission.

In influenza viruses, the posttranslational N-linked glycosylation of the mature HA protein influences virulence and pathogenicity by masking the antigenic epitopes^38^, which changes virus-receptor binding^39^ and modulates the cleavability of the HA precursor protein^40^. Relative to the 2009 canine H3N2 viruses including AS-01/09 (Type I), the H3 HA of the AS-05/12-like viruses (Type II), which were lethal in dogs and readily transmitted between ferrets, has an additional glycosylation at position 63 (lower part of the globular head) and possesses variations in some of the antigenic sites (E1 and D). Although further, detailed molecular studies are needed to elucidate the role of these in virulence, these mutations may indicate continuous evolution of influenza viruses in canine hosts. A total of three viruses, including the AS-05/12 virus, were isolated from individual local pet protection shelters and animal hospitals during the outbreak in the area of Nonsan Korea, and all of the viruses showed genetic characteristics similar to that of the AS-05/12 virus (99.5–100% amino acid homology, data not shown). Although it is not statistically significant, serologic comparison between AS-01/09 and AS-05/12 viruses also indicated differences of at least two-fold (e.g., 320 vs. 640 HI titers). Thus, modifications in HA likely contributed to the further viral adaptation of AS-05/12 and differences in biological characteristics in comparison to previous H3N2 CIV isolates, including the 2009 virus. High pathogenicity is a polygenic trait, and the HA gene most commonly associates with the NS and polymerase genes to produce virulent viruses^41,42^. To elucidate the detailed different pathogenesis of each type of canine viruses, further detailed reverse genetic studies are needed.

One of main objectives of this study was to identify the canine adaptation factors of avian origin H3N2 viruses. To this end, we generated a set of RG viruses in the backbone of LPM91/06 virus with individual gene substitutions from the early canine AS-01/09 virus. Reverse genetics studies revealed that the composition of the NA, NP, and M genes are critical for the canine adaptation of avian LPM91 H3N2 and might contribute to dog-to-dog transmission. In addition, the combined LPM91-Ca-NA/NP/M reassortant virus exhibited marked viral growth and persistence in infected dogs as well as aerosol transmission to contact dogs, similar to the wild-type AS-01/09 virus (Table 3 and Fig. 5). It is noteworthy that the LPM91-Ca-NA/NP/M was the only virus naturally rescued from co-infection in MDCK cell culture, which implies fitness of the avian LPM91 H3N2 virus NA, NP, and M segments. To our knowledge, this study is the first to report successful canine infection of an avian influenza H3N2 virus and to elucidate the potential interspecies transmission factors for canine host adaptation. However, further studies are needed to understand the detailed mechanism of this adaptation and to identify the critical amino acid residues of each internal gene that contribute to the observed phenotypic differences.

Taken together, these results suggest that H3N2 CIVs have successfully adapted and established circulation in Korean canine populations. Furthermore, these viruses are continuously evolving in canine species and thus, are slowly gaining a foothold in mammalian hosts. Importantly, these results underscore the importance of continuous surveillance of CIVs, as it is possible that these viruses could acquire the ability to infect humans.

## Electronic supplementary material

Supplementary Figure S1-1

Supplementary Figure S1-2

Supplementary Figure S1-3

Supplementary Figure S1-4

Supplementary Figure S1-5

Supplementary Figure S1-6

Supplementary Figure S1-7

Supplementary Figure S1-8

Supplementary Figure S2

Supplementary Figure S3

Supplementary figure legends

## Electronic supplementary material

**Supplementary information** The online version of this article (10.1038/s41426-017-0013-x) contains supplementary material.
