# Supplementary figures and images for: Comparison of the virulence and transmissibility of canine H3N2 influenza viruses and characterization of their canine adaptation factors
Source: Emerg Microbes Infect. 2018 Mar 7;7:17. doi: 10.1038/s41426-017-0013-x (PMC5841232; doi:10.1038/s41426-017-0013-x)

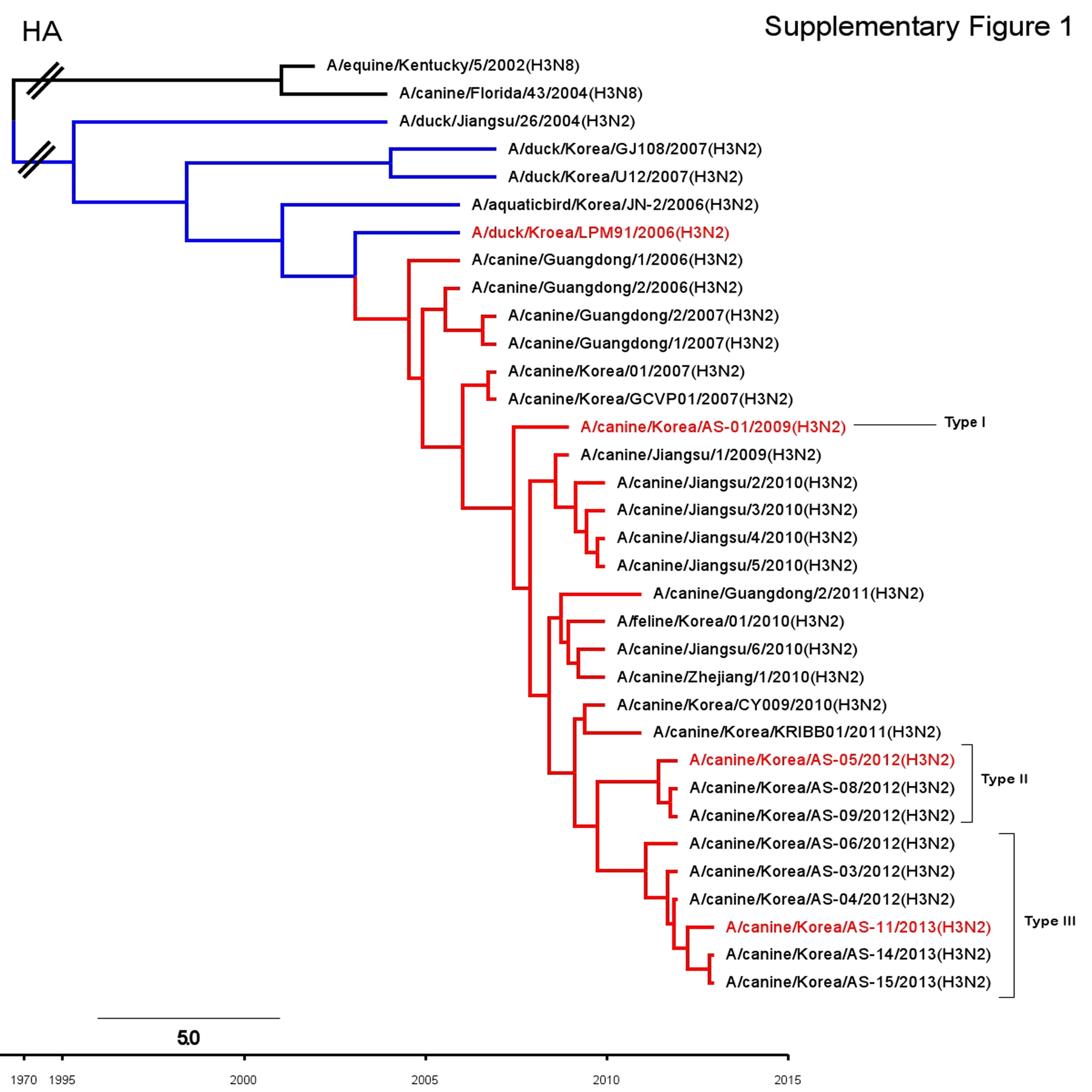

Supplement: Supplementary file 1 — Supplementary Figure S1-1 [file 41426_2017_13_MOESM1_ESM.tif]

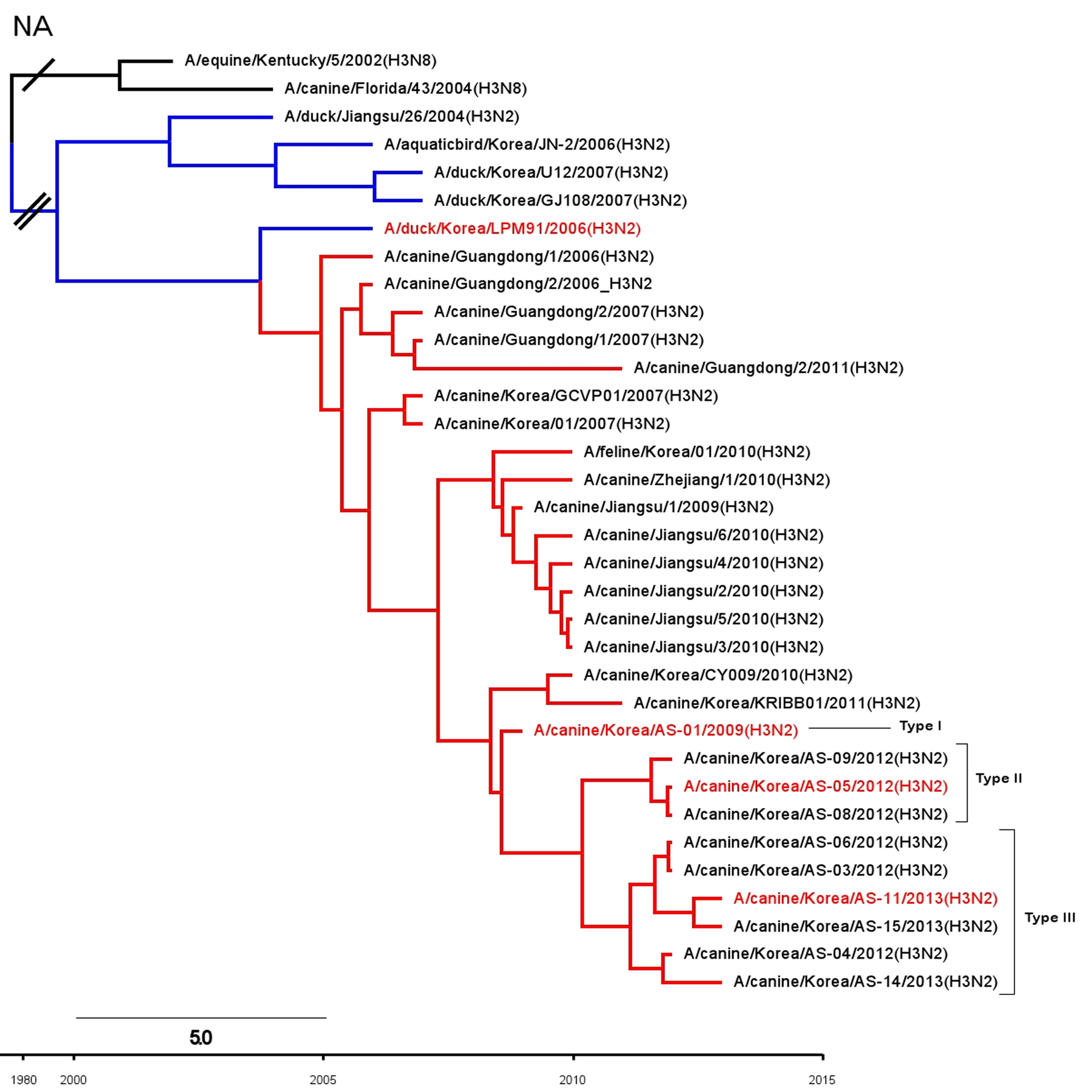

Supplement: Supplementary file 2 — Supplementary Figure S1-2 [file 41426_2017_13_MOESM2_ESM.tif]

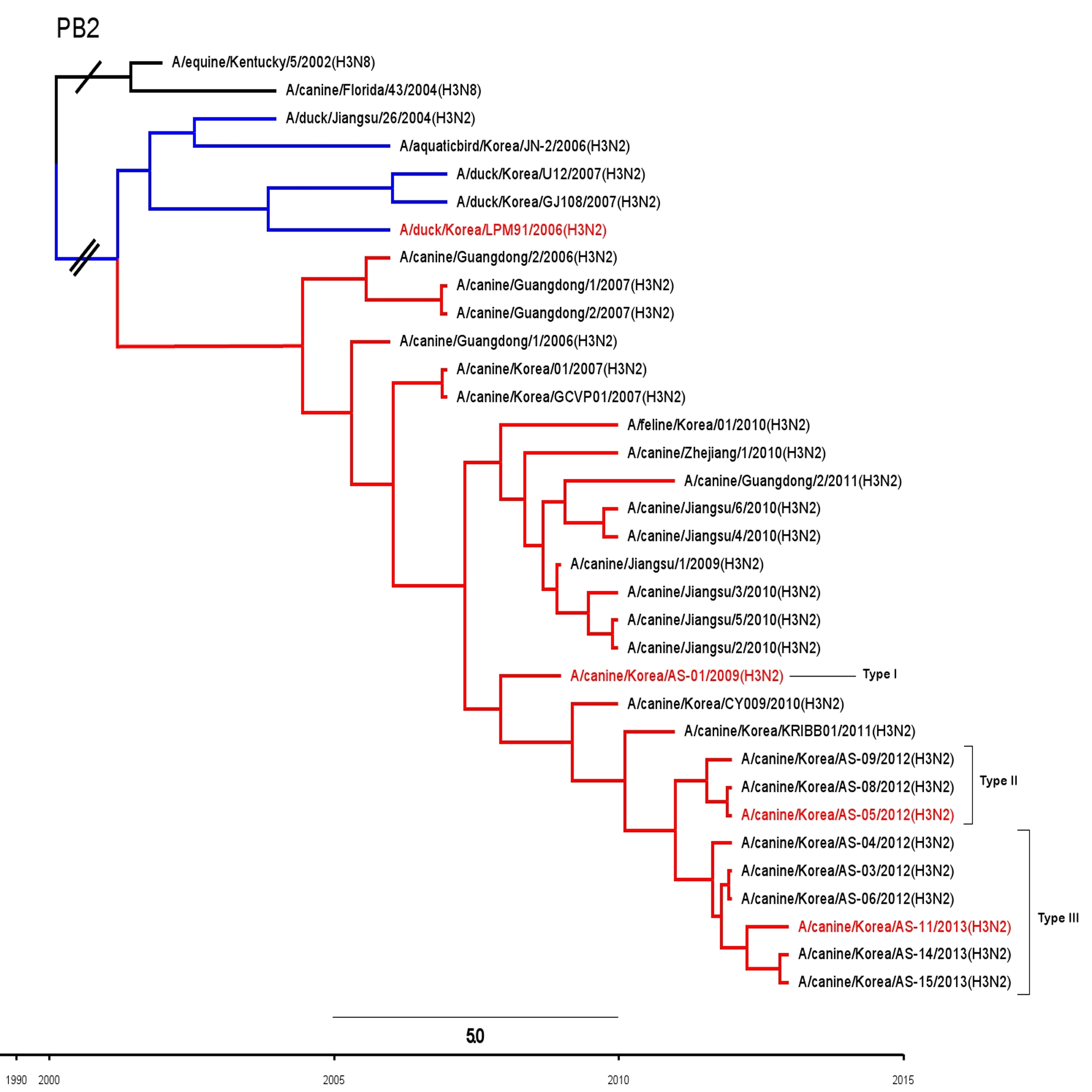

Supplement: Supplementary file 3 — Supplementary Figure S1-3 [file 41426_2017_13_MOESM3_ESM.tif]

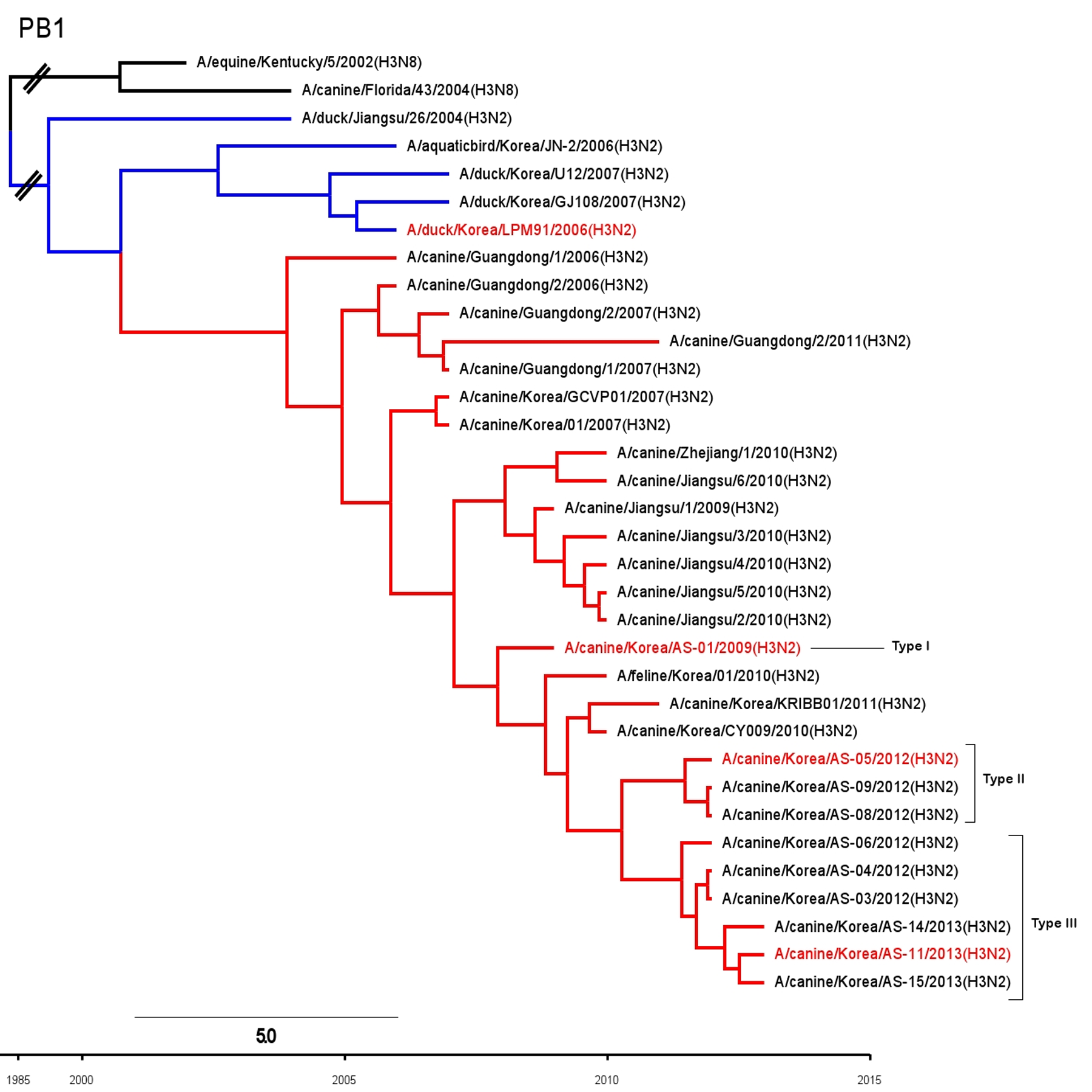

Supplement: Supplementary file 4 — Supplementary Figure S1-4 [file 41426_2017_13_MOESM4_ESM.tif]

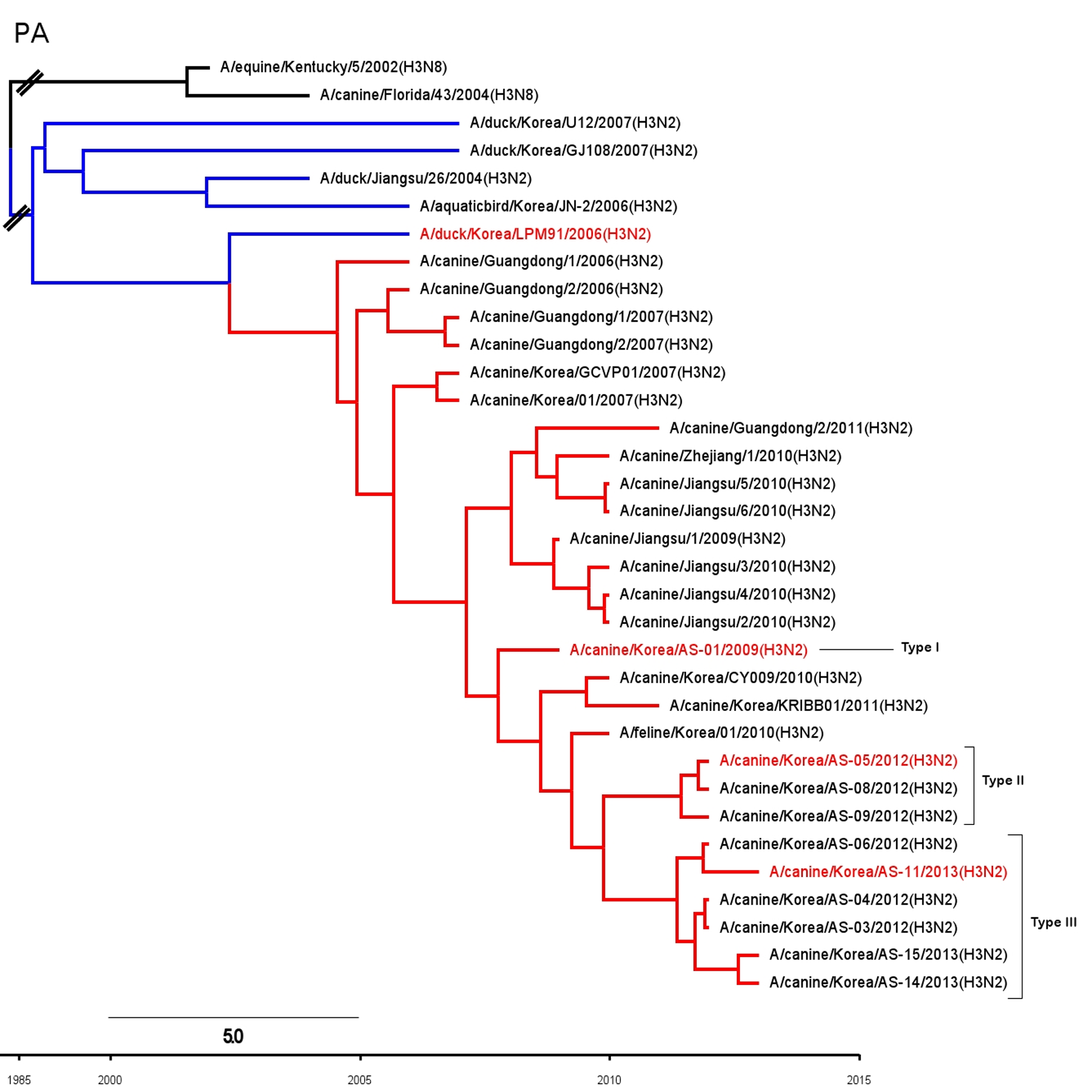

Supplement: Supplementary file 5 — Supplementary Figure S1-5 [file 41426_2017_13_MOESM5_ESM.tif]

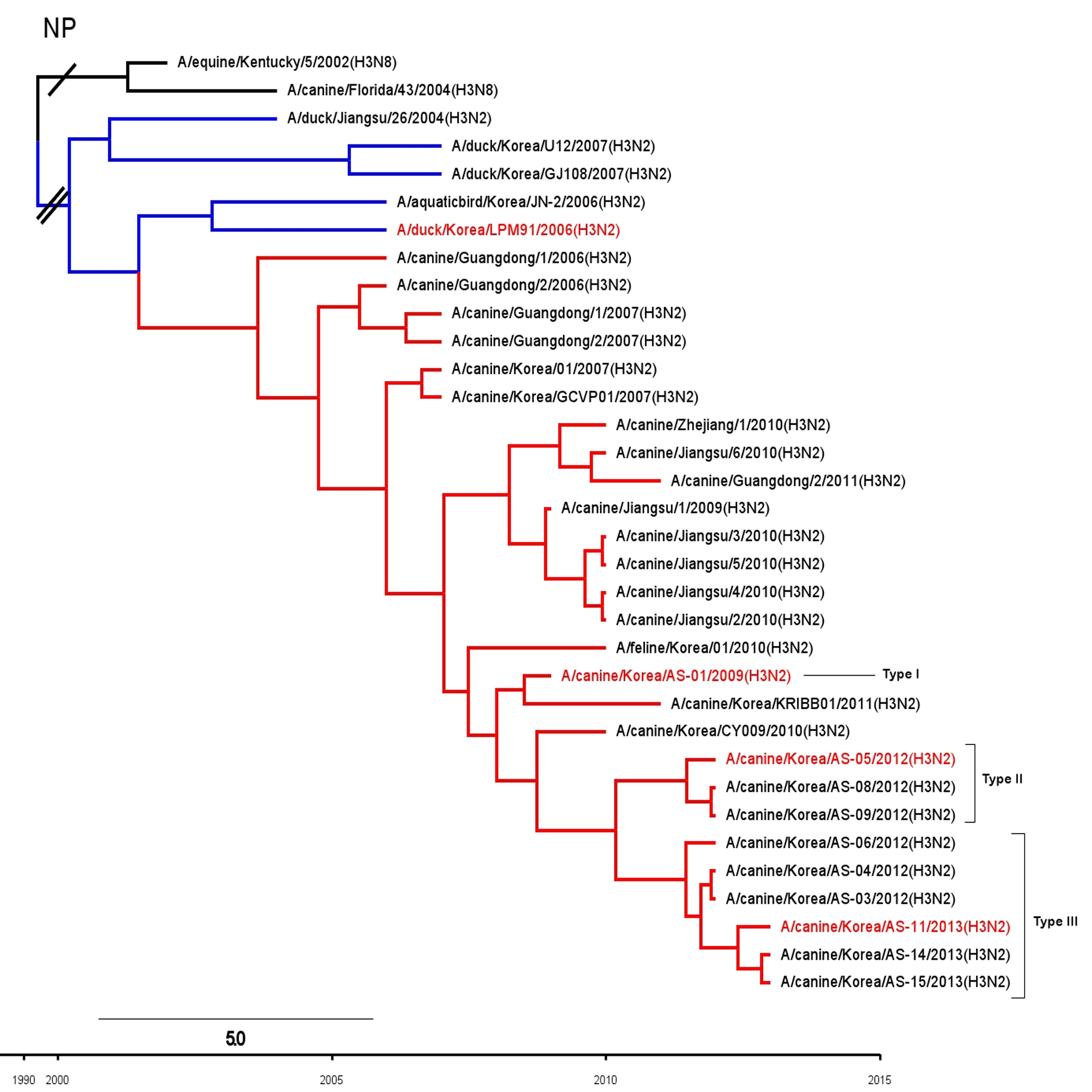

Supplement: Supplementary file 6 — Supplementary Figure S1-6 [file 41426_2017_13_MOESM6_ESM.tif]

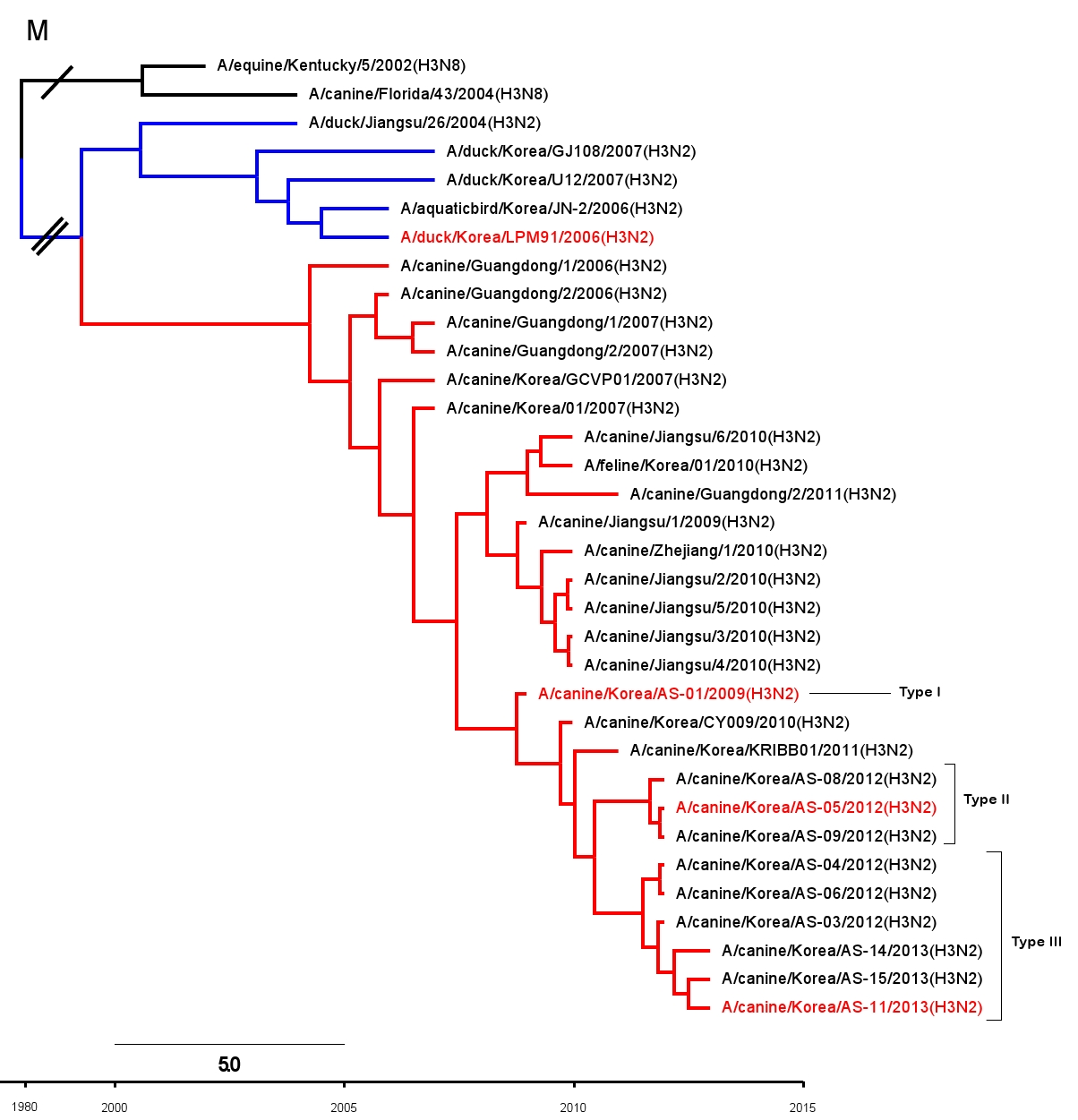

Supplement: Supplementary file 7 — Supplementary Figure S1-7 [file 41426_2017_13_MOESM7_ESM.tif]

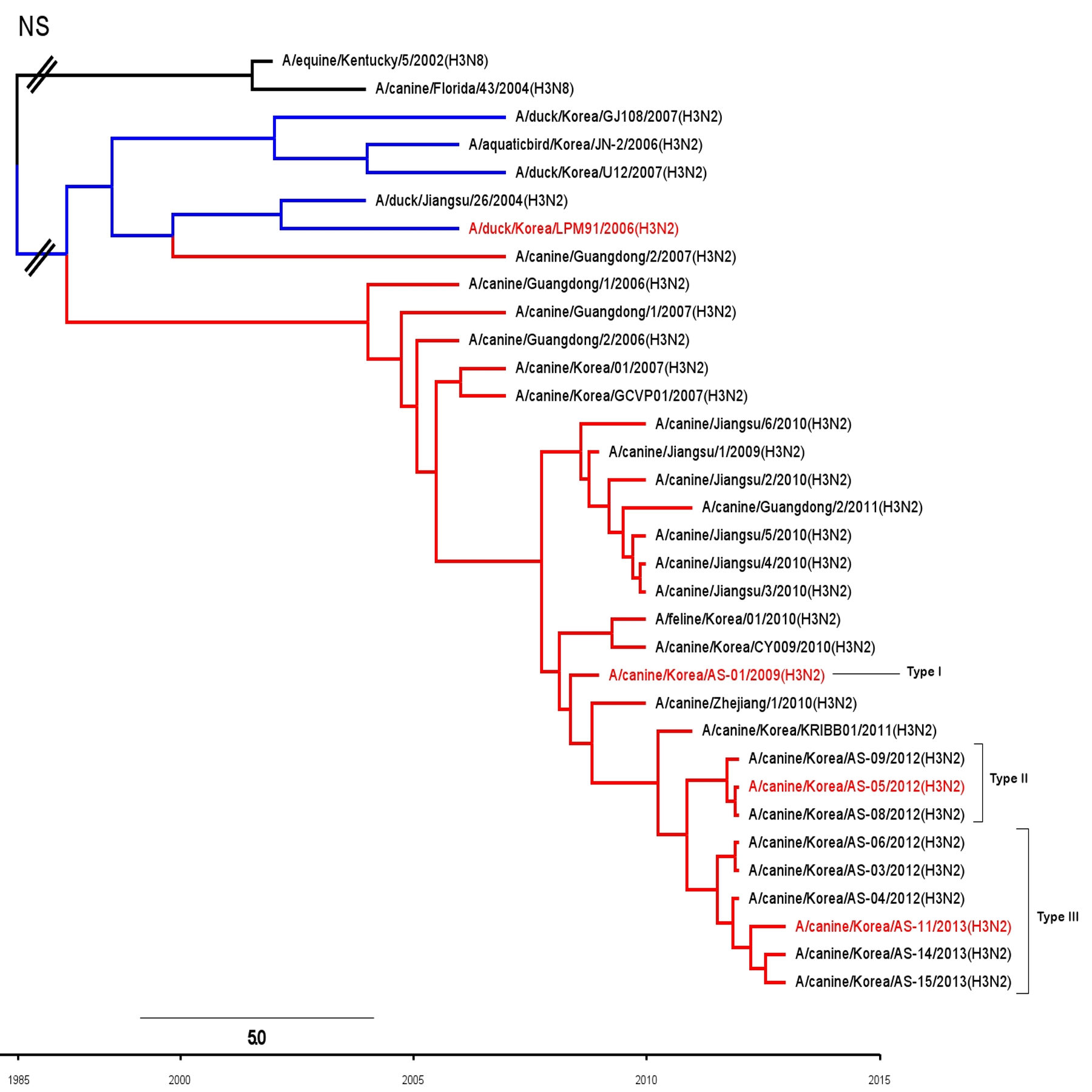

Supplement: Supplementary file 8 — Supplementary Figure S1-8 [file 41426_2017_13_MOESM8_ESM.tif]

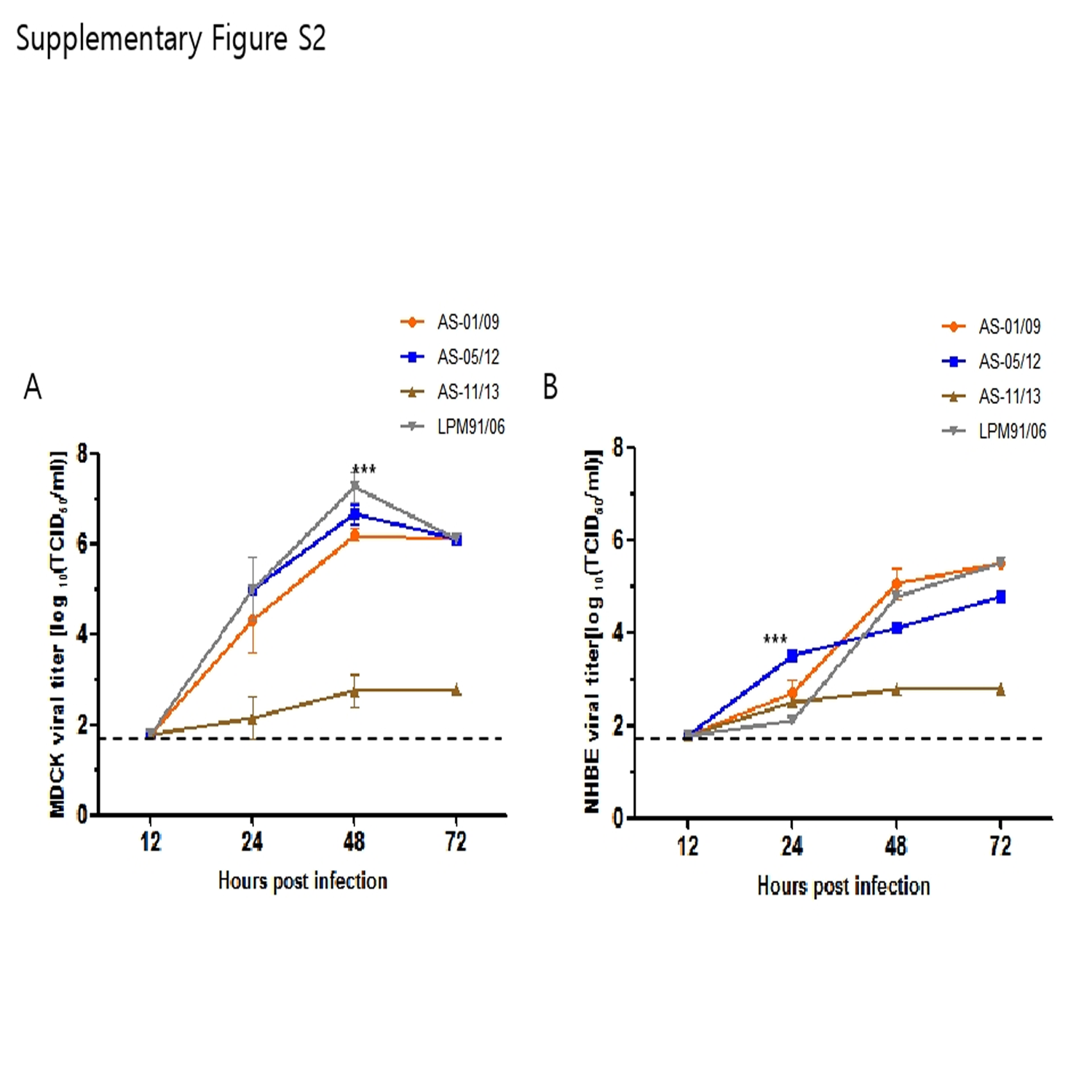

Supplement: Supplementary file 9 — Supplementary Figure S2 [file 41426_2017_13_MOESM9_ESM.tif]

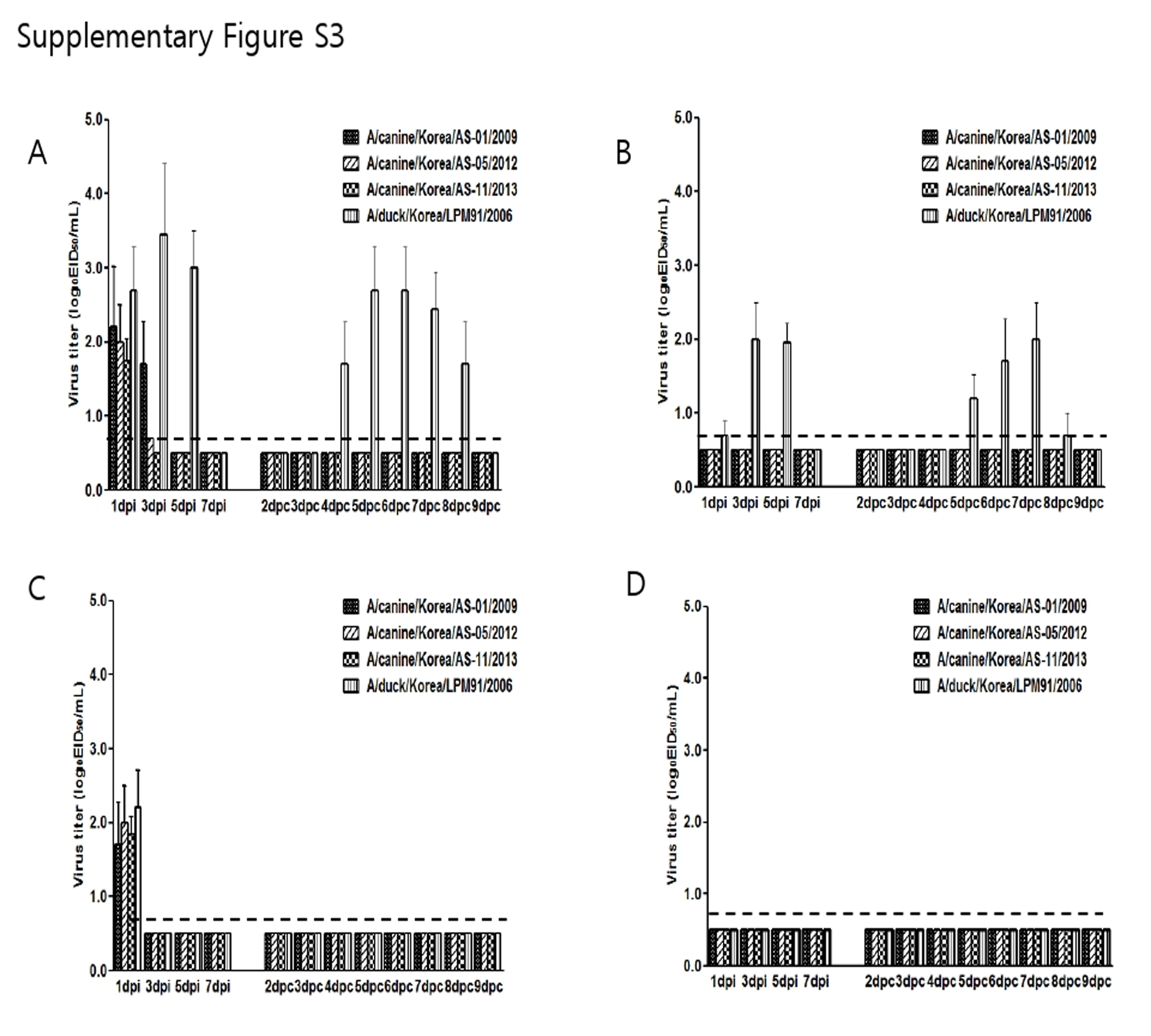

Supplement: Supplementary file 10 — Supplementary Figure S3 [file 41426_2017_13_MOESM10_ESM.tif]
