# Supplementary material for: Comparison of the virulence and transmissibility of canine H3N2 influenza viruses and characterization of their canine adaptation factors
Source: Emerg Microbes Infect. 2018 Mar 7;7:17. doi: 10.1038/s41426-017-0013-x (PMC5841232; doi:10.1038/s41426-017-0013-x)
Supplement: Supplementary file 11 — Supplementary figure legends [file 41426_2017_13_MOESM11_ESM.docx]

**Supplementary Figure S1.** Phylogenetic analysis based on the nucleotide sequences of H3 HA (A), N2 NA (B) and the internal genes PB2, PB1, PA, NP, M, and NS (C to H). Nucleotide sequences were aligned using CLUSTAL_V ^26^. Phylogenetic programs were assigned using the HKY model and the Bayesian evolutionary analysis by sampling trees software package (BEAST) ^27^ and viewed via an Figtree v1.4.3 program (available at: http://tree.bio.ed.ac.uk). The black line indicates North America H3N8 subtype viruses and the blue line indicates the avian-H3N2 subtype viruses in Asia while the red line indicates the canine-H3N2 subtype viruses in Asia. Type I: AS-01/09, Type II: AS-05/12, AS-08/12 and AS-09/12, Type III: AS-11/13, AS-03/12, AS-04/12, AS-06/12, AS-14/13, and AS-15/13. (// : represents the deleted time scale bar since there were large gaps between the North American H3N8 and Asian H3N2 canine viruses).

**Supplementary Figure S2.** Pathogenicity and transmissibility of AS-01/09, AS-05/12, AS-11/13, and LPM91/06 viruses in ferrets. The body temperatures of infected (**A**) and contact (**B**) ferrets were monitored. Groups of three ferrets inoculated with 10^7.0^ EID_50_ of each virus were individually placed adjacent to naïve (contact) ferrets. The nasal-wash titers of both infected and contact ferrets are expressed as log_10_ EID_50_/mL (**C**). The mean viral titers in the brain, trachea, lung, and intestine are shown for each group of ferrets (D). The limit of virus detection was 0.7 log_10_ EID_50_/mL or gram. Statistically significant differences (**P* < 0.05, **: *P* < 0.001, and *** *P* < 0.0001) between each representative canine and AS-01/09 viruses are indicated by * *P* value was determined using one-way ANOVA.

**Supplementary Figure S3.** Pathogenicity and transmissibility of AS-01/09, AS-05/12, AS-11/13, and LPM91/06 viruses as assessed in tracheal and cloacal swabs from ducks (**A** and **B**) and chickens (**C** and **D**). Birds were inoculated with 10^5.5^ EID_50_ of each virus and two naïve counterparts were introduced in the same cage at 1 dpi. The mean viral titers (log_10_ EID_50_/mL) are shown for each group of birds. The limit of virus detection was 0.7 log_10_ EID_50_/mL.
